# Enhanced Carbon monoxide-sensing properties of Chromium-doped ZnO nanostructures

**DOI:** 10.1038/s41598-019-45313-w

**Published:** 2019-06-25

**Authors:** I. Y. Habib, Aimi Asilah Tajuddin, Hafiz Armi Noor, Chee Ming Lim, Abdul Hanif Mahadi, N. T. R. N. Kumara

**Affiliations:** 1 Centre for Advanced Material and Energy Sciences, Universiti Brunei Darussalam, Tungku Link, Gadong, BE1410 Negara Brunei Darussalam; 2 Chemical Sciences, Faculty of Science, Universiti Brunei Darussalam, Jalan Tungku Link, Gadong, BE 1410 Brunei Darussalam

**Keywords:** Nanoparticles, Two-dimensional materials

## Abstract

Low power consumption, fast response and quick recovery times are important parameters for gas sensors performance. Herein, we report the experimental and theoretical studies of ZnO and Cr doped ZnO nanostructures used in low temperature (50 °C) sensors for the detection of CO. The synthesized films were characterized by XRD, UV-Vis, FE-SEM and EDX. The XRD patterns for the ZnO and 0.5 wt% Cr/ZnO films confirm the formation of a single-phase hexagonal wurtzite structure. The reduction of the ZnO optical band gap from 3.12 eV to 2.80 eV upon 0.5 wt% Cr doping is well correlated with the simulation data. The FE-SEM images of the films show spherical morphology with the estimated particle sizes of about ~40 nm and ~ 25 nm were recorded for the ZnO and 0.5 wt% Cr/ZnO films, respectively. Enhanced gas sensing performance is achieved with Cr doping and the sensitivity of ZnO increases from 9.65% to 65.45%, and simultaneously decreasing the response and recovery times from 334.5 s to 172.3 s and from 219 s to 37.2 s, respectively. These improvements in gas sensing performance are due to the reduction in particle size and optical band gap, and an increase in specific surface area.

## Introduction

ZnO as a semiconductor material has been shown to exhibit a wide range of properties for various applications. These properties include; electrical, optical, thermal and structural, which can be utilized depending on the intended area of application^1–3^. In the field of semiconductor metal oxide gas sensor, nanostructured ZnO material has been demonstrated to exhibit remarkable gas sensing properties for variety of gases including reducing, oxidizing, toxic, as well as, flammable gases^4,5^. ZnO nanostructure (NS) shows different sensing mechanisms for oxidizing and reducing gases. In the case of oxidizing gas sensing, such as nitrogen dioxide (NO_2_), the ZnO NS sensor responds by reducing the adsorbed test gas molecules upon surface interaction with the chemisorbed oxygen species leading to the increase in the sensor resistance, with the NO_2_ acting as an electron acceptor as shown in the following equations^4,6^.

1 $${{\rm{NO}}}_{2(g)}+{{\rm{e}}}^{-}\leftrightarrow {{{\rm{NO}}}_{2({\rm{ads}})}}^{-}$$

2 $${{{\rm{NO}}}_{2({\rm{ads}})}}^{-}+{{{\rm{O}}}_{({\rm{ads}})}}^{-}+2{{\rm{e}}}^{-}\leftrightarrow {{\rm{NO}}}_{({\rm{g}})}+2{{\rm{O}}}^{2-}$$

On the contrary, the surface reaction mechanism involved with reducing gases such as H_2_S, CH_4_ and H_2_, is based on the interaction of the test gas molecules with the chemisorbed oxygen species, which is accompanied by the release of trapped electrons absorbed by the chemisorbed oxygen species back to the ZnO conduction band, and eventually leading to the decrease in the sensor resistance as shown in the equations below^5–7^.

3 $$2{{\rm{H}}}_{2}{{\rm{S}}}_{({\rm{g}})}+3{{{\rm{O}}}_{2({\rm{ads}})}}^{-}\leftrightarrow 2{{\rm{H}}}_{2}{{\rm{O}}}_{({\rm{g}})}+2{{\rm{SO}}}_{2(g)}+3{{\rm{e}}}^{-}$$

4 $${{\rm{H}}}_{2(g)}+{{{\rm{O}}}_{({\rm{ads}})}}^{-}\leftrightarrow {{\rm{H}}}_{2}{{\rm{O}}}_{({\rm{g}})}+{{\rm{e}}}^{-}$$

5 $${{\rm{CH}}}_{4({\rm{g}})}+4{{{\rm{O}}}_{({\rm{ads}})}}^{-}\leftrightarrow {{\rm{CO}}}_{2}+2{{\rm{H}}}_{2}{{\rm{O}}}_{({\rm{g}})}+2{{\rm{e}}}^{-}$$

It is imperative to note that the most distinctive difference between the reducing and oxidizing gas molecules, is their surface reaction behaviors. As highlighted by Tee *et al*., the reducing gases are usually not adsorbed at the oxides surface like the oxidizing gas molecules, instead they only react with the chemisorbed oxygen species, as summarized by Eqs (3)–(5).

However, most of the gas sensing reactions involving ZnO NS occur at temperatures between 200–400 °C. This is due to a typical property of ZnO NS having low specific surface area, wide band gap and low porosity^6,8^. The operations at high temperatures bring added challenges, whereby, more thermal electrons are introduced, and the test gas also exhibits higher kinetic energy. In light of the above issues, several dopants have been utilized to engineer the microstructure of ZnO NS, and to reduce the operating temperature of the device. Xu *et al*. has successfully doped ZnO NS with La^3+^ at high temperature, and this produces an increase in the specific surface area and eventually leading to the enhancement of acetone gas sensing sensitivity^9^. In addition, ZnO NS doped with Mn is found to be effective for detecting a wide range of reducing substances, such as acetone, ethanol and acetic acid at temperatures above 400 °C^10^. This remarkable sensitivity obtained from the above study is attributed to the presence of lower ionization energy of Mn as compared to the ZnO NS material, and the ability of the Mn to reduce the activation energy of the surface chemisorbed gases.

For CO gas sensing, the ZnO doped with In^3+^ system has been found to improve the response time of the CO detection at 300 °C. This is attributed to the reduction of the energy band gap, as well as, the ability of the In^3+^ to act as donor species leading to the formation of more adsorption sites, which mainly consist of In atoms and oxygen vacancies^8^.

Zhang *et al*. has successfully doped Cr precursor into ZnO nanorods by hydrothermal method. The ZnO–Cr system was used in the gas sensing studies of reducing and toxic gas such as ammonia; flammable and volatile liquids such as acetone and ethanol; corrosive liquid such as acetic acid; and organic solvent such as dimethyl formamide, at operating temperature of 300 °C^11^. From the literature review, it is evidenced that the enhanced gas sensing response of ZnO for various test gases with different dopants occurs at temperatures above 200 °C. Therefore, the objective of this study is to achieve similar or better gas sensing sensitivities at relatively lower temperature.

In pursuing the objective of this study, investigation based on density functional theory (DFT) with transition metals (Sc, Ti, V, Cr, Mn and Fe) doped ZnO nanocage as proposed by Aslanzadeh *et al*. is considered^12^. The purpose is to investigate the electronic conductivity, optical, and gas sensing properties of ZnO and transition metals doped ZnO in CO atmosphere^12^. Cr and V doping significantly improve the sensitivity and electrical conductivity of the cluster when compared to Mn, Fe, Sc and Ti dopants.

This paper presents the experimental work for Cr doped ZnO NSs gas sensor and it is complimented by DFT calculations for the ZnO (Zn_12_O_12_) and Cr-ZnO (CrZn_11_O_12_) nano-cages. The experimental results show the improved performance of the sensor, and the material characterization (XRD, UV-Vis, FE-SEM and EDX) methods were used to study the properties of the material. Sol-gel technique was used to synthesize the ZnO, and Cr doped ZnO and this method is shown to provide excellent doping control and consistent quality re-production. The optimal percentage by weight of the Cr doping for the sensor is also determined in this study.

## Materials and Method

### Computational methods

The molecules used for the DFT calculations are constructed using Spartan-10. The molecules structures are spherical nano-cages (Zn_12_O_12_ and Cr doped Zn_11_O_12_). The structures are constructed from a combination of hexagon and tetragon cells. The optimization of geometries and energy calculations are determined using B3LYP, where 6–31 G(d) is used as the basis set for the Gaussian program. This basis set is one of the most commonly used for ZnO based nanoclusters (NCs)^12,13^.

The E_g_ (optical ban gap);

6 $${{\rm{E}}}_{{\rm{g}}}={{\rm{E}}}_{{\rm{L}}{\rm{U}}{\rm{M}}{\rm{O}}}-{{\rm{E}}}_{{\rm{H}}{\rm{O}}{\rm{M}}{\rm{O}}}$$

where ${{\rm{E}}}_{{\rm{LUMO}}}\,{\rm{and}}\,{{\rm{E}}}_{{\rm{HOMO}}}$ are the energies of the lowest unoccupied molecular orbital (LUMO) and that of the highest occupied molecular orbital (HOMO), respectively.

### Experimental details

Zinc acetate di-hydrate [Zn (CH_3_COO)_2_. 2H_2_O, MW = 219.49] was utilized as precursor to synthesize ZnO, and Chromium nitrate nonahydrate [Cr(NO_3_)_3_.9H_2_O, MW = 400.21] was used as precursor for the dopant material. Methanol [CH_3_OH, MW = 32.04] and Cetyltrimethylammonium bromide, CTAB [(C_16_H_33_)N(CH_3_)_3_]Br, MW = 364.45] were used as solvent and stabilizing agent, respectively. All chemicals and reagents were used from Merck Germany and of analytical grade, and they were used without further purification.

### Sol-gel synthesis of ZnO (Pure) and 0.5 wt% Cr/ZnO Nanopowders

The sol-gel method employed in the present study is similar to that reported by Basyooni *et al*.^14^. 1.54 g of Zn(CH_3_COO)_2_.2H_2_O was weighed and transferred into a 100 ml beaker. 14 ml of CH_3_OH was then added to prepare the 0.5 M solution, followed by the addition of 1 ml of 0.5 M CTAB to control the shape and size of the ZnO material. The mixture was then magnetically stirred in a hotplate at 60 °C for 2 h to form a clear homogeneous solution (alcosol). To obtain the Cr doped ZnO sols, specific amount of Cr(NO_3_)_3_.9H_2_O precursor was added to the above initial starting materials to form the corresponding 0.5 wt% Cr/ZnO. The mole ratio of ZnO to CTAB was set at 1:1. The solution mixtures have pH values of about 6. The resulting alcosols were then aged for about 48 h to gel at room temperature, and then further dried at 70 °C for 10–12 h in a hot plate to evaporate the excess methanol. It is then grounded into powders and centrifuged 5 times in hexane at 7000 rpm for 10 min. The cleaned powders were then dried overnight at RT and further dried in a preheated oven for 24 h at 100 °C to evaporate the excess hexane and other organic residuals. The resulting nanopowders were designated as ZnO (Pure) and 0.5 wt% Cr/ZnO corresponding to the undoped and 0.5 wt% Cr doped ZnO NSs respectively.

### Fabrication of ZnO (Pure) and 0.5 wt% Cr/ZnO films

The ZnO and 0.5 wt% Cr/ZnO paste were prepared by manual mixing of 0.2 g each of either ZnO (Pure) or 0.5 wt% Cr/ZnO NSs with 2 ml ethanol and 1 drop of Triton-X in a very smooth agate mortar and pestle until the desired viscosity was obtained. The paste was then deposited on a 5-μm gold interdigitated electrode (5 μm-Au-IDE) using doctor-blade method of deposition. The resulting film (active area ~50 mm^2^) was then heated in a preheated oven for 1 h at 60 °C and subsequently calcined in a furnace at 400 °C for 2 h under at a ramping rate of 1 °C/min to remove the binders, excess ethanol, as well as, to stabilize the metal oxide sensing layer^15^. The corresponding average film thickness as estimated by a Surface Profiler (Model; ALPHA STEP IQ) was found to be ~20 μm.

### Films characterizations

The structural properties of the ZnO (Pure) and 0.5 wt% Cr/ZnO films were determined using Shimadzu X-ray diffractometer XRD-7000 with Cu Kα radiation source (λ = 1.54060 Å) at 2θ range between 20–80° and a sampling rate of 5°/min. The average crystallite sizes of the ZnO (Pure) and 0.5 wt% Cr/ZnO films were estimated using the known Scherrer equation. The film morphologies were determined using field emission scanning electron microscope (FE-SEM; JEOL, model: (JSM-7610F)), whereas the chemical compositions were analyzed using energy dispersive X-ray spectroscopy (EDX). Cary 500 UV-Vis-NIR Spectrophotometer was utilized to measure the ultraviolet visible light absorptions of the films across the wavelength range between 200–800 nm.

### Gas sensing system and measurements

**Figure 1:**
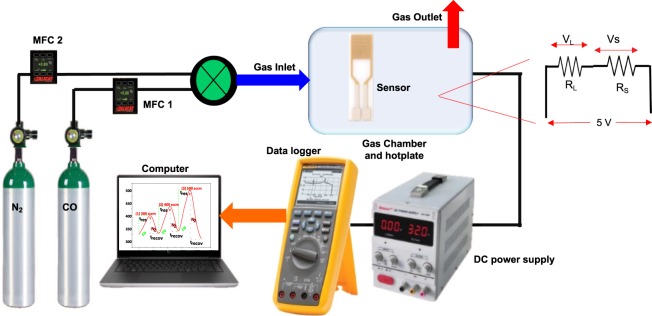
A schematic diagram for the experimental set-up of the gas sensing system.

Figure 1 shows the schematic diagram of the system used to measure the gas-sensing performance of the fabricated samples. The setup design and sensor parameters calculations such as sensor resistance and sensitivity were based on the published literature reported by Basyooni *et al*.^14^. The fabricated sensor was placed in a gas chamber. The gas inlet that flowed into the gas chamber was controlled by a mass flow controller (MFC). The load resistance, R_L_ (430 kΩ) was used, and input voltage, V_i_ (5 V) was applied from a DC power source. R_L_ was connected in series with the sensor device. A Fluke Digital Multimeter version 289 was used to measure the sensor output signal (V_out_). The sensor resistance was calculated using the following equation;

7 $${{\rm{R}}}_{{\rm{S}}}=\frac{{{\rm{V}}}_{{\rm{i}}}-{{\rm{V}}}_{{\rm{out}}}}{{{\rm{V}}}_{{\rm{out}}}}{{\rm{R}}}_{{\rm{L}}}$$

where R_s_ = sensor resistance, V_i_ = input voltage, V_out_ = output voltage, R_L_ = load resistance. The sensitivity (sensor response), S is given by;

8 $${\boldsymbol{S}}( \% )=\frac{{{\rm{R}}}_{{\rm{CO}}}-{{\rm{R}}}_{{\rm{N}}2}}{{{\rm{R}}}_{{\rm{N2}}}}\times 100$$

where R_CO_ denotes the sensor resistance recorded at CO atmosphere and R_N2_ denotes the sensor resistance recorded at nitrogen atmosphere.

The sensors were tested in the presence of N_2_ as the carrier gas and CO as the test gas across a wide range of flow rate (300–500 sccm) at 50 °C. The test began by heating the sensor material to 50 °C and left to stabilize for 1 hour. This also helps to remove the gaseous impurities such as CO, hydrocarbons and humidity in the chamber^15^. The duration of the test was set to a maximum of 10 min for a complete cycle. It is important to note that the sensor material is only subjected to a single gas atmosphere (CO or N_2_) at a time.

## Results and Discussion

### Computational analysis

**Figure 2:**
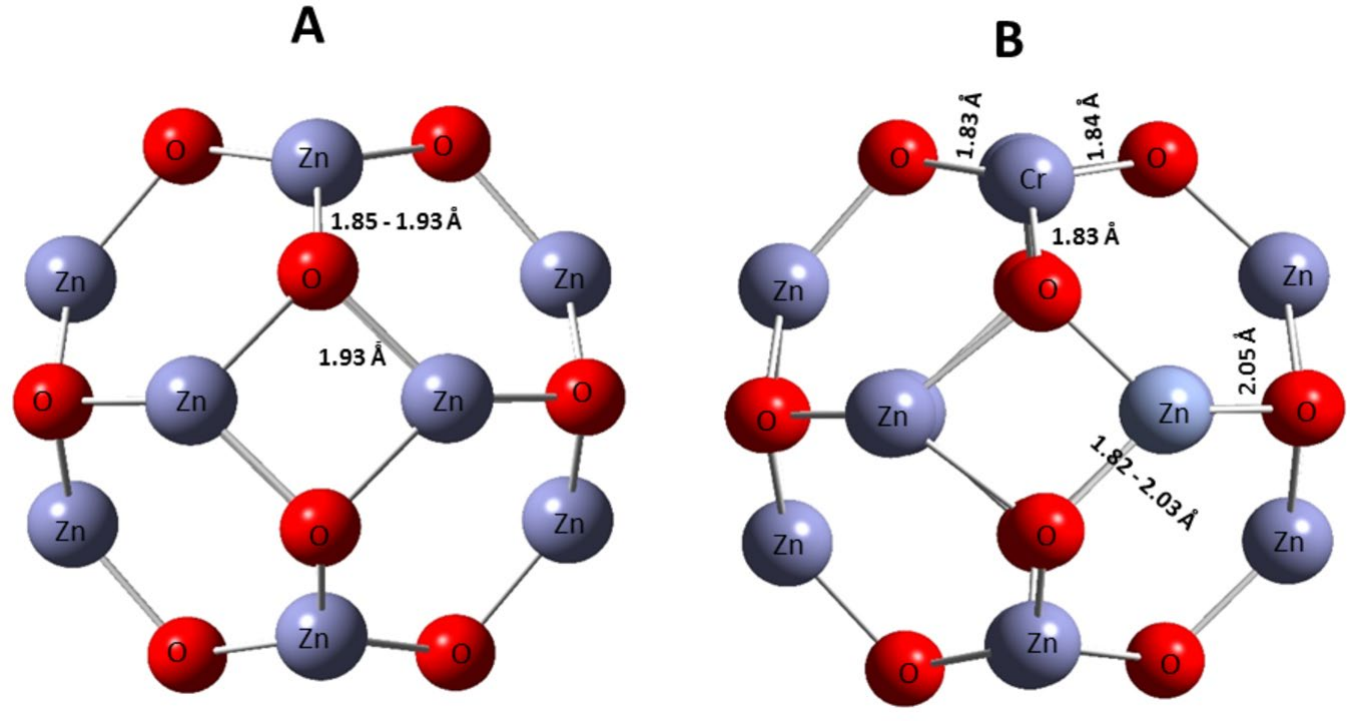
Optimized structures of (**A**) Zn_12_O_12_ nano-cage, (**B**) Cr doped Zn_11_O_12_ nano-cage.

The optimized geometry of the pristine Zn_12_O_12_ nano-cage is shown in Fig. 2(A). The figure shows a Zn_12_O_12_ cage containing 12 Zn atoms and 12 O atoms forming 4 tetragons and 8 hexagons. The Zn - O bond length in the tetragons are about 1.93 Ǻ with the bond angles within the range of 86.9–92°. The hexagonal structures have bond lengths in the range of 1.85–1.93 Å, which are formed between Zn and O atoms. The inner angles of the hexagons range from 112–126°. The calculated energy levels; HOMO, LUMO and the energy band gaps are shown in Table 1. The optimized geometry of the Cr doped Zn_11_O_12_ nano-cage is shown in Fig. 2(B). In the tetragon structure within the Cr doped Zn_11_O_12_ cage, it is found that the oxygen bonded to the chromium (Cr–O) has a bond length of 1.83 and 1.84 Å and the Zn–O bond length lies between 1.82–2.03 Å. The angles produced by the tetragons lie between 112.60–130°. The oxygen - chromium - oxygen (O–Cr–O) bond angle is estimated to be 130°. The hexagon structure of Cr doped Zn_11_O_12_ has a bond length of 1.83 Å with both O bonded to the Cr atoms and the (Zn–O) bond length is found to be 2.05 Å while its bond angle ranges from 78.0–95.20° and the angle between the Cr and the other oxygen atoms were found to be 78°. These simulated Zn–O bond length values were found to be similar to that obtained by Aslanzadeh *et al*., which was in the range of 1.92–1.99 Å^12^. The calculated levels for HOMO, LUMO and the energy band gaps are also illustrated in Table 1.

**Table 1:** Energies of HOMO and LUMO levels and band gap of Zn_12_O_12_ and Cr/Zn_11_O_12_.

System	E_homo_ (eV)	E_lumo_ (eV)	E_gap_ (eV)
Zn_12_O_12_	−7.00	−2.86	4.14
Cr/Zn_11_O_12_	−4.45	−2.77	1.68

The HOMO and LUMO energy levels are −7.00 eV and −2.86 eV respectively, generating E_g_ value of about 4.14 eV for Zn_12_O_12_. After doping Cr with Zn_11_O_12_, the HOMO and LUMO energy values of the Cr doped ZnO nanocluster are to −4.45 eV and −2.77 eV for the HOMO and LUMO levels, respectively. The energy band gap of the pristine Zn_12_O_12_ has been significantly decreased from 4.14 to 1.68 eV upon Cr doping, as seen in Table 1. Cr doping has changed the microstructural, electrical and optical properties of the Zn_12_O_12_ nano-cage. The relationship between the band gap and the change in conductivity is shown in the following equation^12^.

9 $${\rm{\sigma }}={{\rm{AT}}}^{3/2}\exp (-\frac{{{\rm{E}}}_{{\rm{g}}}}{2{\rm{kT}}})$$

where σ is the conductivity, A is a constant, T is the temperature and k is the Boltzmann’s constant.

This equation shows that the conductivity changes exponentially with the energy band gap. The conductivity is typically used as an indication of the sensitivity of the nano-cages toward varieties of chemicals. There are other factors that could lead to a better gas sensing response through doping a suitable dopant into the pristine Zn_12_O_12_ nano-cage. Firstly, is the change in activation energy, which can easily enhance the gas sensor response and recovery times as well as its selectivity^13^. As gas approaches the surface of a sensor material at a given temperature, it absorbs energy and suddenly becomes activated. Once activated, it will then react with the particles absorbed at the surface of the sensor material, which could be expressed using the following reaction:

10 $${\rm{r}}={\rm{C}}\,\exp (-\frac{{{\rm{E}}}_{{\rm{a}}}}{{\rm{kT}}})$$

where r denotes the rate of reaction and C denotes the pre-exponential factor.

Reaction rate constant increases as activation energy decreases. Hence, the rate at which a sensor material achieves its response increases exponentially by lowering the activation energy. Moreover, the kinetic energy and adsorption desorption rates of a gas molecules increase with increase in temperature, which counters the increase in sensitivity, hence temperature variations play a vital role in a gas sensing study. Another advantage of doping is that, it generates oxygen vacancy and increases the conductivity of a metal oxide semiconductor. Oxygen vacancies are considered important reactive agents for many adsorbates. Hence, the type of point defect influences many surface reactions. Oxygen vacancy could act as a direct adsorption site, as well as electron donor. Furthermore, oxygen vacancies could act as nucleation centers for small metal clusters. Doping a cluster will have a significant effect for metal oxides, which are dominated by defect chemistry via oxygen vacancy^14^.

### XRD analysis

**Figure 3:**
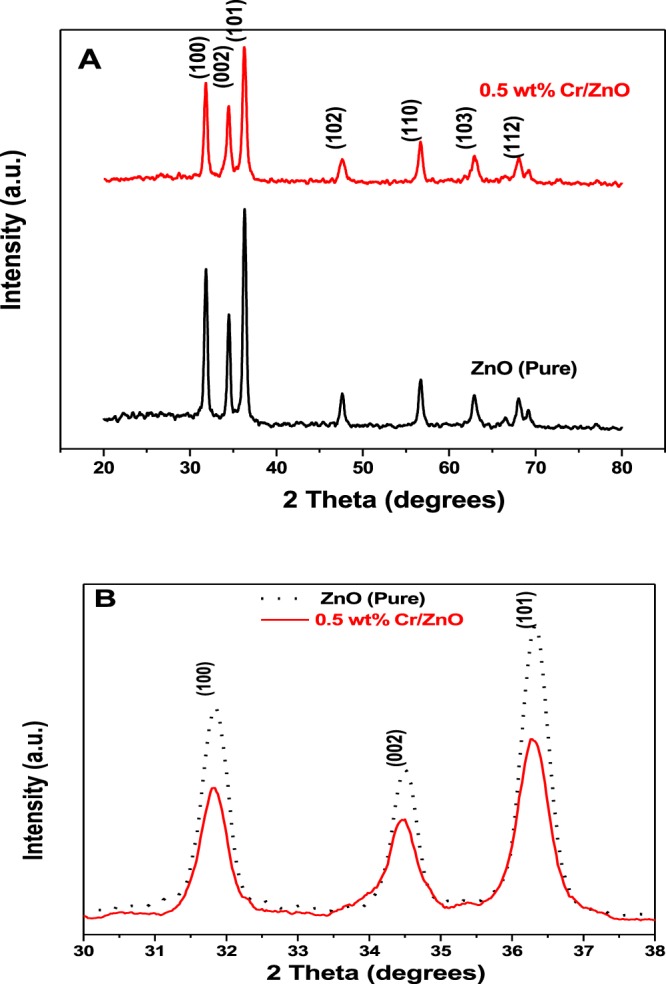
(**A**) X-ray diffraction patterns for the ZnO (Pure) and 0.5 wt% Cr/ZnO films and (**B**) X-ray diffraction peaks of the most intense reflections for the ZnO (pure) and 0.5 wt% Cr/ZnO films.

The X-ray diffraction of the ZnO (Pure) and 0.5 wt% Cr/ZnO films are carried out to investigate their structural properties. Figure 3(A) illustrates the X-ray diffraction patterns of the ZnO (Pure) and 0.5 wt% Cr/ZnO films. These reflections correspond to single phase hexagonal wurtzite structure of ZnO material (JCPDS cards no. 89-0510)^3^. The presence of the sharp peaks indicated high crystallinity of the materials.

However, Cr doping inhibits the crysOtal growth by lowering the peak intensities^16^. It is observed that Cr doping did not change the diffraction peaks positions of the ZnO (Pure) film as illustrated in Fig. 3(B), suggesting the absence of any significant lattice shift on the ZnO wurtzite structure upon Cr doping. Similar diffraction pattern was also reported from literature^17^. The complete absence of additional peak due to Cr compounds implies its remarkable dispersion as shown in Fig. 3(A). It could be argued that Cr atoms are present in the ZnO crystal lattice by replacing some of the Zn atoms in the crystal lattice as the ionic radius of Cr^3+^ (0.615 Å) is smaller than that of Zn^2+^ (0.74 Å) while that of Cr^2+^ (0.73 Å) is very similar to that recorded for Zn^2+^ ^16^. The crystallite sizes for the samples are calculated using the known Scherrer’s equation^18^.

11 $${D}=\frac{\kappa \,{\rm{\lambda }}}{\,{\rm{\beta }}\,\cos \,{\rm{\theta }}}$$

where D stands for the average crystallite size (nm), к is a dimensionless shape factor, with a value close to unity (0.9), λ = 1.54060 Å, the wavelength of the monochromatic incident Cu Kα radiation and β is the full width at half maximum intensity of the respective peaks measured in radians and θ is the diffraction angle.

The calculated crystallite sizes are 49.80 nm and 20.04 nm corresponding to ZnO (pure) and 0.5 wt% Cr/ZnO films, respectively, as presented in Table 2. It could be seen that the average crystallite size of the ZnO (pure) film is decreased significantly from 49.80 to 20.04 nm upon doping with 0.5 wt% Cr.

**Table 2:** The XRD data for the ZnO (pure) and 0.5 wt% Cr/ZnO films; the texture coefficient (TC), the crystallite size (D), the lattice parameter (a, c and volume of the unit cell V), u parameter, and Zn-O bond length (L).

Material	D (nm)	Lattice parameters			u	L (Å)	TC		
		a(Å)	c(Å)	V(Å^3^)			100	002	101
ZnO (pure)	49.80	3.2449	5.1975	47.3945	0.3799	1.9747	0.68	1.11	1.21
0.5 wt% Cr/ZnO	20.04	3.2387	5.2007	47.2397	0.3793	1.9724	0.76	1.20	1.04

The ZnO structural parameters are calculated using Eqs (12–16) ^14^. The ZnO lattice parameters ‘a’ and ‘c’ are given by Eq. (12).

12 $$\frac{1\,}{{{\rm{d}}}^{2}}=\frac{4}{\,3}\frac{({{\rm{h}}}^{2}+{{\rm{k}}}^{2}+{\rm{hk}})}{{{\rm{a}}}^{2}}+\frac{{{l}}^{2}}{{{\rm{c}}}^{2}}$$

where d, is the inter planar spacing while the h, k and l are the miller indices.

The corresponding values of the lattice parameters are presented in Table 2. The volume, V, of the unit cell of the hexagonal wurtzite structure of the ZnO, is obtained from Eq. (13).

13 $${\rm{V}}=\frac{\sqrt{3}}{\,2}{{\rm{a}}}^{2}{\rm{c}}$$

The calculated volumes of the unit cells are included in Table 2. The volume of ZnO is slightly decreased from 47.3945–47.2397 Å^3^ upon the introduction of 0.5 wt% Cr precursor and this indicates a slight lattice contraction leading to the formation of lattice defects. The position parameter ‘u’, which is an important variable in calculating the Zn–O bond length is given by Eq. (14).

14 $${\rm{u}}=\frac{{{\rm{a}}}^{2}}{3{{\rm{c}}}^{2}}$$

The resulting values of the calculated u parameter where also listed in Table 2. Note that the value of ‘a’ obtained for ZnO (pure) decreases with 0.5 wt% Cr doping. The ZnO bond length, L is obtained from Eq. (15).

15 $${\rm{L}}={[\frac{{{\rm{a}}}^{2}}{3}+{(\frac{1}{2}-{\rm{u}})}^{2}{{\rm{c}}}^{2}]}^{1/2}$$

The calculated values of L are presented in Table 2. The values of Zn–O bond length recorded for ZnO (Pure) and 0.5 wt% Cr/ZnO are 1.9747 Å and 1.9727 Å, respectively. The slight decrease in the Zn–O bond length could be associated with the slight decrease in the values of the lattice parameters “a” and “c”. The experimental values are similar to the simulated results of about 1.85–1.96 Å and 2.05 Å recorded for ZnO, and Cr doped ZnO nanocages, respectively.

The texture coefficient, TC, is another important parameter that is commonly used to quantitatively predict the preferential crystal orientation towards a specific direction. In the present study, the occurrence of the calculated TC values >1 at 002 and 101 planes for the ZnO (Pure) film, while the occurrence of TC value >1 is only at the 002 plane for the 0.5 wt% Cr/ZnO film (Table 2). The 002 plane as a preferential grains orientation for a composite material has shown to be a strong potential for remarkable gas sensor response^14^. The TC values of the ZnO (Pure) and 0.5 wt% Cr/ZnO films are given by using Eq. (16);

16 $${T}{{C}}_{({\rm{hkl}})}=\frac{{{I}}_{{\rm{r}}({\rm{hkl}})}}{{{\rm{N}}}^{-1}\sum {n}\,{{I}}_{{\rm{r}}({\rm{hkl}})}}$$

where n is the number of diffraction peaks, I_r(hkl)_ is the ratio between the measured relative intensity of XRD planes (hkl) to its standard intensity taken from the American Mineralogist Crystal Structure Database (AMCSD-0005203) for a Zincite material, and N is the reflection number.

The specific surface areas (S_s_) for the ZnO and the 0.5 wt% Cr/ZnO films is given by the following BET equation^19^.

17 $${{\rm{S}}}_{{\rm{s}}}=\frac{{\rm{6}}}{{{\rm{D}}}_{{\rm{t}}}\ast {{\rm{D}}}_{{\rm{XRD}}}}$$

where D_t_ is the theoretical density (D_tZnO_ = 5.606 g/cm^3^) and D_XRD_ is the average crystallite size calculated using Eq. (8).

The values of the specific surface areas are presented in Table 3 with the 0.5 wt% Cr/ZnO film having a higher specific surface area of about 53.41 m^2^/g than the ZnO (Pure) film of about 21.49 m^2^/g. This higher surface area obtained for the 0.5 wt% Cr/ZnO film could be attributed to the smaller average crystallite size value as shown in Table 2. For hexagonal wurtzite structural material such as ZnO, the in-plane stress (σ) of the films could be calculated using the biaxial strain model according to following equation^20^;

18 $${\rm{\sigma }}=4.5\times {10}^{11}\frac{{{\rm{C}}}_{{\rm{o}}}-{\rm{C}}}{{{\rm{C}}}_{{\rm{o}}}}$$

where C and Co (AMCDS, 5.2038 Å) are the experimental and standard lattice parameter, respectively.

**Table 3:** The In-plane stress (σ), the specific surface area (S_s_), the micro-strain (ε) and dislocation density (δ) for the ZnO (pure) and 0.5 wt% Cr/ZnO films.

Material	Stress, σ × 10^9^	S_s_ (m^2^/g)	Micro-strain, ε	Dislocation density, δ × 10^−3^ (lines/nm^2^)
ZnO (pure)	0.54	21.49	0.13	0.40
0.5 wt% Cr/ZnO	0.27	53.41	0.33	2.49

The calculated values were also presented in Table 3. The micro-strain (**ε**), is defined by the equation below^21^.

19 $${\boldsymbol{\varepsilon }}=\frac{{\rm{\beta }}\,\tan \,{\rm{\theta }}}{4}$$

where β is the full width at half maximum intensity of the 3 most intense XRD peaks illustrated in Fig. 3B.

The calculated values of micro-strain of the films are listed in Table 3. The dislocation density (δ) is calculated using the following equation^22^.

20 $${\rm{\delta }}=\frac{1}{{{\rm{D}}}^{{\rm{2}}}}$$

where D is the average crystallite size. The corresponding D values of the films are also listed in Table 2.

The calculated values of both the micro-strain and the dislocation density are both shown in Table 3. It is clearly observed that 0.5 wt% Cr/ZnO film exhibits lower stress value indicating good stability upon calcination and having higher values of micro-strain and dislocation density than the ZnO (Pure). These variations indicate lattice deterioration of the ZnO microstructure and hence defects formation. This phenomenon could be ascribed to the inherent property of Cr ions to be utilized as both iso-valent and alio-valent dopant in ZnO semiconductor material. Similar characteristics of lattice strain inducement is also evident when Sb^3+^ ions are doped into ZnO microstructure. The ionic radii of Sb^3+^ (0.078 nm) and ZnO (0.074 nm) are similar, and this substitution produces stacking fault defect in the Sb-ZnO nanocomposites^23^.

### Films morphology

**Figure 4:**
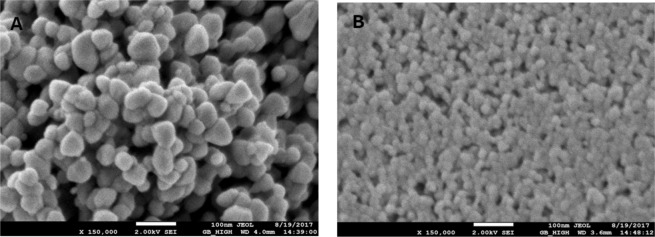
FE-SEM images (scale; 100 nm, mag; x 150,000) for (**A**) ZnO (pure) (**B**) 0.5 wt% Cr/ZnO.

The morphologies of the ZnO (Pure) and 0.5 wt% Cr/ZnO films are determined using field emission scanning electron microscope (FE-SEM). Figure 4(A,B), shows the surface morphology images of the films on a scale of 100 nm and 150, 000 magnification. Both the ZnO (Pure) and the 0.5 wt% Cr/ZnO have spherical morphology with particle sizes in the nanometer range. The ZnO (pure) film particle’s surface appeared to be densely packed with some of the particles lying on top of the others leading to agglomeration, as shown in Fig. 4(A). The films are shown to be composed of highly nanocrystalline particles, which are similar to those reported by Strauma *et al*.^24^. Cr doping is found to change the orientation of the particles by the increase in the packing density, as well as, the inter-growth leading to a modified inter-particles network, as shown in Fig. 4(B). Figure 4(B) also clearly shows an improved agglomeration, and an amorphous like region is induced in the Cr doped ZnO film. Similar particles orientation is also observed upon Mn doping in ZnO NS^24,25^. The distribution of the particle’s diameter for ZnO (Pure) is found to be ~ 40 nm, and this decreases to ~25 nm upon Cr doping, as shown in Fig. 4. Such decrease in the particle size of ZnO upon Cr doping enhances the material’s surface area and its morphological and catalytic properties^26^.

### Energy dispersive X-ray spectroscopy (EDX)

**Figure 5:**
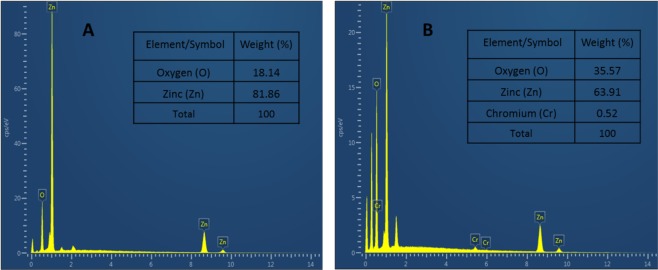
EDX spectra of (**A**) ZnO (Pure) and (**B**) 0.5 wt% Cr/ZnO NSs films.

The chemical compositions of the ZnO (Pure) and 0.5 wt% Cr/ZnO films have been determined using energy dispersive X-ray spectroscopy (EDX). The EDX spectra for the films containing an inset showing the percent composition of the chemical constituents is presented in Fig. 5. Both films show the presence of Zn and O atoms, which are the main building blocks of the ZnO material, as shown in Fig. 5(A,B). The presence of Cr in the ZnO NS is also evident as stated in Fig. 5(B).

### UV-Vis spectroscopy

**Figure 6:**
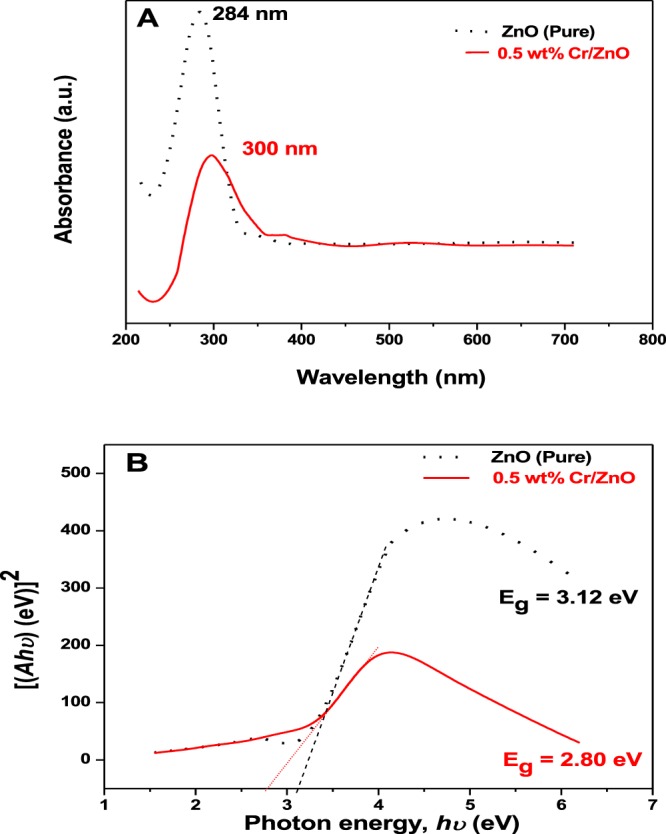
(**A**) Absorbance spectra and (**B**) Band gap energy plots for the ZnO (pure) and 0.5 wt% Cr/ZnO NS films.

The UV-Vis analyses of the ZnO (Pure) and 0.5 wt% Cr/ZnO NS films are carried out with UV-Vis spectrophotometer within the 200–800 nm range (Fig. 6(A)). The absorption peak for ZnO (pure) was recorded at 284 nm. The occurrence of this intense absorption peak is a good indication of the material’s potential to be employed in an electronic applications such as gas sensors, photocatalysis and solar cells^27–30^. On the other hand, the absorption wavelength obtained for the 0.5 wt% Cr/ZnO film is at 300 nm, (Fig. 6(A)), indicating a red-shift upon Cr doping. The obtained absorbance values are used to calculate the direct energy band gap of the films using Tauc equation^18,31,32^.

21 $${\rm{\alpha }}\mathrm{hv}=A(\mathrm{hv}-{\mathrm{Eg})}^{\frac{1}{2}}$$

where α, A, hʋ and E_g_ correspond to the absorption coefficient, constant, photon energy (eV), and optical band gap, respectively.

Extrapolation has been carried out to obtain the E_g_ values on the photon energy axis (x-axis). Figure 6(B) depicts the optical band gaps of the ZnO (Pure) and 0.5 wt% Cr/ZnO films in eV. It is observed that the optical band gap value extrapolated for ZnO (pure) is found to be 3.12 eV, which is relatively lower when compared to the reported value for bulk ZnO material which is 3.37 eV^6,23^. Yogamalar and Bose also reported relatively lower optical band gap values of about 2.93, 2.97 and 2.99 eV for hydrothermally grown ZnO nanorods^33^. Despite the relatively low optical bandgap values recorded by ZnO (pure) in the present study, its value is also found to have significantly dropped to 2.80 eV upon doping with 0.5 wt% Cr. This observation is in good agreement with the optical band gap decrease of the pristine Zn_12_O_12_ nanocage from 4.14 to 1.68 eV upon Cr doping, as demonstrated by the simulation data shown in Table 1.

Several literatures have also reported the enhancement of ZnO NS upon the introducing of an impurity atom, though the decrease in the optical band of any support material may not necessarily be feasible in the presence of unsuitable dopant. For example, the ZnO semiconductor optical band gap is measured to be 3.17 eV as reported by Hendi and Alorainy, but their band gap value was found to have increased from 3.17 to 3.27 eV upon loading the ZnO microstructure with Cu concentration ranging from 0.1 to 5 wt %^34^.

This decrease in band gap upon doping was also observed by Hosseini *et al*. where doping with Ag has led to the decrease in the ZnO optical band gap from 3.25 to 3.18 eV^35^. The decrease in the optical band gap energy upon Cr doping unto the ZnO NS could have resulted in the formation of defects and higher flow of electrons from the valence band to the conduction band of ZnO material, which is an inherent property of n-type semiconductor material, thus resulting in the increase in the electronic conductivity of the sensor material^16^.

### Dynamic response curve analysis

**Figure 7:**
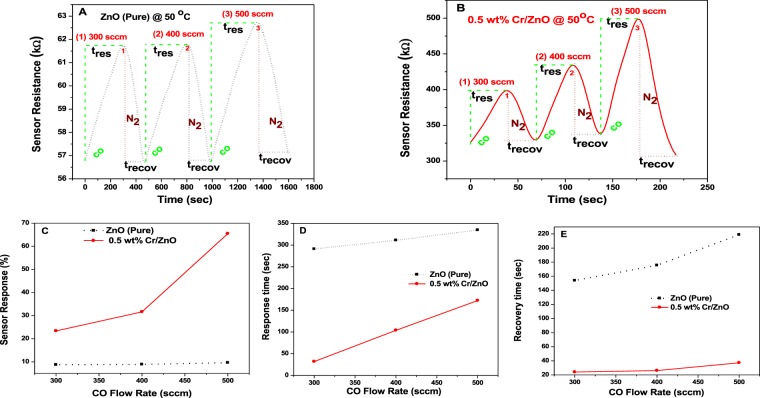
The dynamic response curves for (**A**) ZnO (pure) and (**B**) 0.5 wt% Cr/ZnO sensor resistance vs. the detection time; (**C**) The sensor response (%) as a function of CO concentration in sccm (**D**) Response time (t_res_) and (**E**) Recovery time (t_recov_) of ZnO (Pure) and 0.5 wt% Cr/ZnO sensors vs. CO concentration in sccm.

The gas sensor test in the present study has been carried out in the presence of N_2_ as the carrier gas and CO as the test gas across a wide range of flow rate (300–500 sccm) at 50 °C. The schematic of the gas sensor system is shown in Fig. 1. The dynamic response curves show the relationship between the resistance, response and recovery times for the ZnO (Pure) and 0.5 wt% Cr doped ZnO sensors (Fig. 7(A,B)). When the sensor material is subjected to the test gas, its resistance increases due to the interaction between the test gas and the surface adsorbed oxygen molecules. Conversely, the resistance of the sensing material decreases when the sensor material is introduced to the carrier gas atmosphere while switching off the test gas. Such decrease in the sensor resistance could be ascribed to the reduction in the electron depletion region^36^. Therefore, the sensor resistance in a gas sensing experiment is one of the crucial parameters to measure gas sensing sensitivity. The gas sensor resistance is calculated using Eq. (7).

In all the measurements recorded in the present study, the sensor resistance increased to the maximum saturation point as CO was introduced and then decreased as CO was switched back to N_2_ atmosphere, where the resistance reverted to the baseline value as shown in Fig. 7(A,B). The resistance values can be used to measure the sensor response or sensitivity (%). The sensitivity is calculated using Eq. (8). It could be observed that the sensitivity increases linearly with an increase in the CO flow rates from 300–500 sccm as shown in Fig. 7(C). The values of the sensitivities recorded for the ZnO (Pure) and the 0.5 wt% Cr/ZnO sensors are presented in Table 4.

**Table 4:** Values of sensor response (sensitivity %), response time, and recovery time *vs* CO concentration.

Material	Flow rate (sccm)	Sensor Response (%)	Response time, t_res_ (sec)	Recovery time t_recov_ (sec)
ZnO (Pure)	300	8.77	290.90	154.00
	400	8.90	311.00	175.50
	500	9.65	334.50	219.00
0.5 wt% Cr/ZnO	300	23.38	32.00	24.10
	400	31.62	103.8.00	26.20
	500	65.45	172.3.00	37.20

The observed sensitivity range of ZnO (pure) across the measured flow rates is between 8.77–9.65%. This low sensitivity of the gas sensing behavior of ZnO (Pure) sensor could be attributed to its low surface area and wide optical band gap. Tee *et al*., also added that the occurrence of charge carriers recombination process at the ZnO surface after CO oxidation could be another important factor that leads to the low sensitivity of ZnO^1^. The sensitivity recorded in the presence of 0.5 wt% Cr/ZnO sensor across the CO flow rate range (300–500 sccm) is between 23.38–65.53%. This significant increase in the sensitivity exhibited by 0.5 wt% Cr/ZnO sensor is ascribed to its higher surface area and lower optical band gap, stronger synergistic coupling of electronic and chemical properties, as well as, stronger grain orientation towards the 002 plane than the ZnO (Pure) film as shown in Tables 2 and 3 ^5,9,14^. Hjiri *et al*. also reported similar gas sensing behavior based on Ga-doped ZnO sensor for CO detection^37^.

It is also important to note that the best gas sensing sensitivity for both the ZnO (Pure) and 0.5 wt% Cr/ZnO sensors occur at the highest flow rate (500 sccm) as shown in Table 4. The time taken for the sensor resistance to achieve its maximum value in the present study is taken as the response time (t_res_), whereas the time taken for the sensor resistance to drop down to the baseline resistance is referred to as recovery time (t_recov_). The t_res_ and t_recov_ for the ZnO (Pure) sensor occur in the range 290.90–334.50 s and 154.00–219.00 s, respectively. Similarly, the t_res_ and t_recov_ for the 0.5 Cr wt%/ZnO sensor occur in the range 32.00–172.3 s and 24.10–37.20 s, respectively. The t_res_ and t_recov_ for both the ZnO (Pure) and 0.5 wt% Cr/ZnO sensors vary linearly with increase in CO flow rates, as shown in Fig. 7(D,E). It is worth noting that doping with Cr precursor induces a substantial decrease in the t_res_ and t_recov_ of the ZnO (Pure) sensor to approximately 2 and 6 folds respectively (Table 4). This increase in gas sensing sensitivity toward CO gas in the present study is similar to the gas sensing study reported by Basyooni *et al*. on Na doped ZnO gas sensor for sensing CO_2_ gas^14^.

### Gas sensing mechanism

A typical gas sensing behavior of a semiconducting material is based on the strong interaction between the test gas molecules and the adsorbed oxygen species at the surface of the sensing material. The adsorption of the target gas at the surface of the ZnO material depends on the type of the chemisorbed oxygen species (O_2_^−^, O^−^, O^2−^) previously adsorbed at the ZnO surface. It is reported by Kimiagar *et al*. that the occurrence of defects such as Zn interstitials and oxygen vacancies on ZnO surface enable oxygen molecules in ambient environment to be adsorbed easily due to the differences in chemical potential energy^38^. Therefore, the adsorbed oxygen molecules could capture one or two electrons from the ZnO conduction band and subsequently ionized into O^−^, O_2_^−^ or O_2_^2−^ species through chemisorption. Since the chemisorption process is temperature dependent and the temperature used to carry out gas sensing test in the present study is virtually low, thus nullifying the effect of di-ionic molecular or atomic oxygen species (O_2_^2−^ or O^2−^). As a result, a single molecular anionic oxygen species (O_2_^−^) is more likely to be present, hence the following equations could be applied^5^.

22 $${{\rm{O}}}_{2({\rm{g}})}\leftrightarrow {{\rm{O}}}_{2({\rm{ads}})}$$

23 $${{\rm{O}}}_{2({\rm{ads}})}+{{\rm{e}}}^{-}\leftrightarrow {{{\rm{O}}}_{2({\rm{ads}})}}^{-}$$

24 $${\rm{CO}}+{{{\rm{O}}}_{2({\rm{ads}})}}^{-}\leftrightarrow {{\rm{CO}}}_{2}+{{\rm{e}}}^{-}$$

The gas sensing mechanism begins, when the sensor material is first exposed to air, gaseous oxygen molecules (O_2 (g)_) undergo chemisorption on its surface to become chemically adsorbed (O_2 (ads)_) and they become negatively charged species (O_2 (ads)_^−^) by capturing electrons from the ZnO conduction band as shown in Eqs (22–23). The test gas (CO) is then reacted with the chemisorbed oxygen species according to Eq. (24). The electrons released in Eq. (24) are then returned to the ZnO conduction band and are therefore responsible for decreasing the sensor resistance^6,39^. It is also worth mentioning that conducting a gas sensing test at a very low temperature (25–100 °C) could result to the formation of fewer oxygen species because of the low thermal energy involved, since oxygen species are mostly thermally stable due to their chemisorption on the surface of the metal oxide^6^. Therefore, the role played by the Cr dopant could be highly commendable as it significantly reduces the activation energy of the mentioned reactions, making the reaction possible to be carried out at lower temperature, and eventually leading to enhanced sensor sensitivity. The above mechanisms show that the gas sensing process strongly depends on the surface property of the ZnO semiconducting material, as well as, its interaction between the potential oxygen species. Therefore, the 0.5 wt% Cr/ZnO sensor is found to be more sensitive towards CO detection than the ZnO (Pure) sensors, as demonstrated by the presence of lower values obtained for the dislocation density and micro-strain, hence, the increase in its sensitivity. Other factor that leads to the enhanced sensitivity of the 0.5 wt% Cr/ZnO sensor could be assigned to the presence of donor defects which could lead to apparently more chemisorbed and ionized oxygen species and therefore increasing the electron concentrations, and consequently leading to the increase in the sensor resistance^39^. This could be described using the following proposed Kroger Vink Notation.25where a dot in the above equation indicates a positive charge. From Eq. (22), the substitution of Zn^2+^ with Cr^3+^ into ZnO crystal lattice releases one free electron to the system. These free electrons increases the density of surface adsorbed oxygen species, as well as, the system conductivity^11^. The remarkable sensitivity and selectivity demonstrated by the 0.5 wt% Cr/ZnO sensor explains the relevance of this material for sensing other toxic gases such as ammonia, hydrogen sulfide, nitrogen dioxide and phosgene. To our knowledge, this is the first time a theoretical and experimental studies based on the undoped and Cr doped ZnO nanostructure films were incorporated for the low temperature gas sensing of CO.

## Conclusion

ZnO (Pure) and 0.5 wt% Cr/ZnO NS sensors have been successfully synthesized by sol-gel method. The films were characterized by XRD, UV-Vis, FE-SEM and EDX. The XRD patterns for the ZnO (Pure) and 0.5 wt% Cr/ZnO NS films confirm the formation of a single-phase hexagonal wurtzite structure of ZnO material. The calculated average crystallite sizes are 49.80 and 20.04 nm, for the ZnO (Pure) and 0.5 wt% Cr/ZnO films, respectively. The UV-Vis absorption wavelength shifted from 284 nm to 300 nm, and the optical band gap is shown to have decreased from 3.12 eV to 2.84 eV upon Cr doping. These results correlated well with the substantial optical band gap decrease as observed in the DFT simulations which have shown a change from 4.14 eV to 1.68 eV with Cr doping. The particle sizes obtained from FE-SEM also correlated well with the calculated average crystallite sizes for both films. The gas sensing properties toward CO for the ZnO (Pure) and 0.5 wt% Cr/ZnO sensors occurred at the flow rate range between 300–500 sccm at 50 °C. The sensitivity, t_res_ and t_recov_ for both the ZnO (Pure) and 0.5 wt% Cr/ZnO sensors vary linearly with the increase in CO flow rates. This enhanced low temperature gas sensing performance exhibited by the 0.5 wt% Cr/ZnO sensor could be attributed to the lower particle size and reduced optical band gap.
